# Supplementary figures and images for: Temporal discounting in adolescents and adults with Tourette syndrome
Source: PLoS One. 2021 Jun 18;16(6):e0253620. doi: 10.1371/journal.pone.0253620 (PMC8213148; doi:10.1371/journal.pone.0253620)

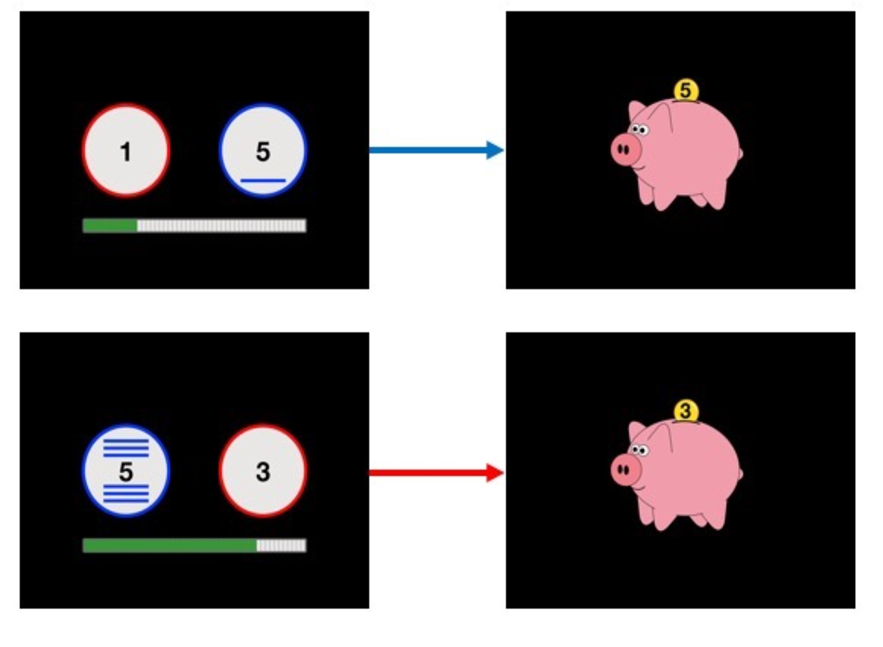

Supplement: S1 Fig — The blue circle depicts the LL reward (in cents) that participants will receive if they wait. How long they have to wait is indicated by blue lines, i.e. one blue line = 10s wait, six blue lines = 60s wait. The red circle indicates how much the participant will receive if they move on to the next trial immediately (0–4 cents). Participants received feedback about the amount earned after every trial (piggy bank). The green bar below the two circles indicates how many trials the participant has already finished. LL, larger but later. (TIF) [file pone.0253620.s001.tif]

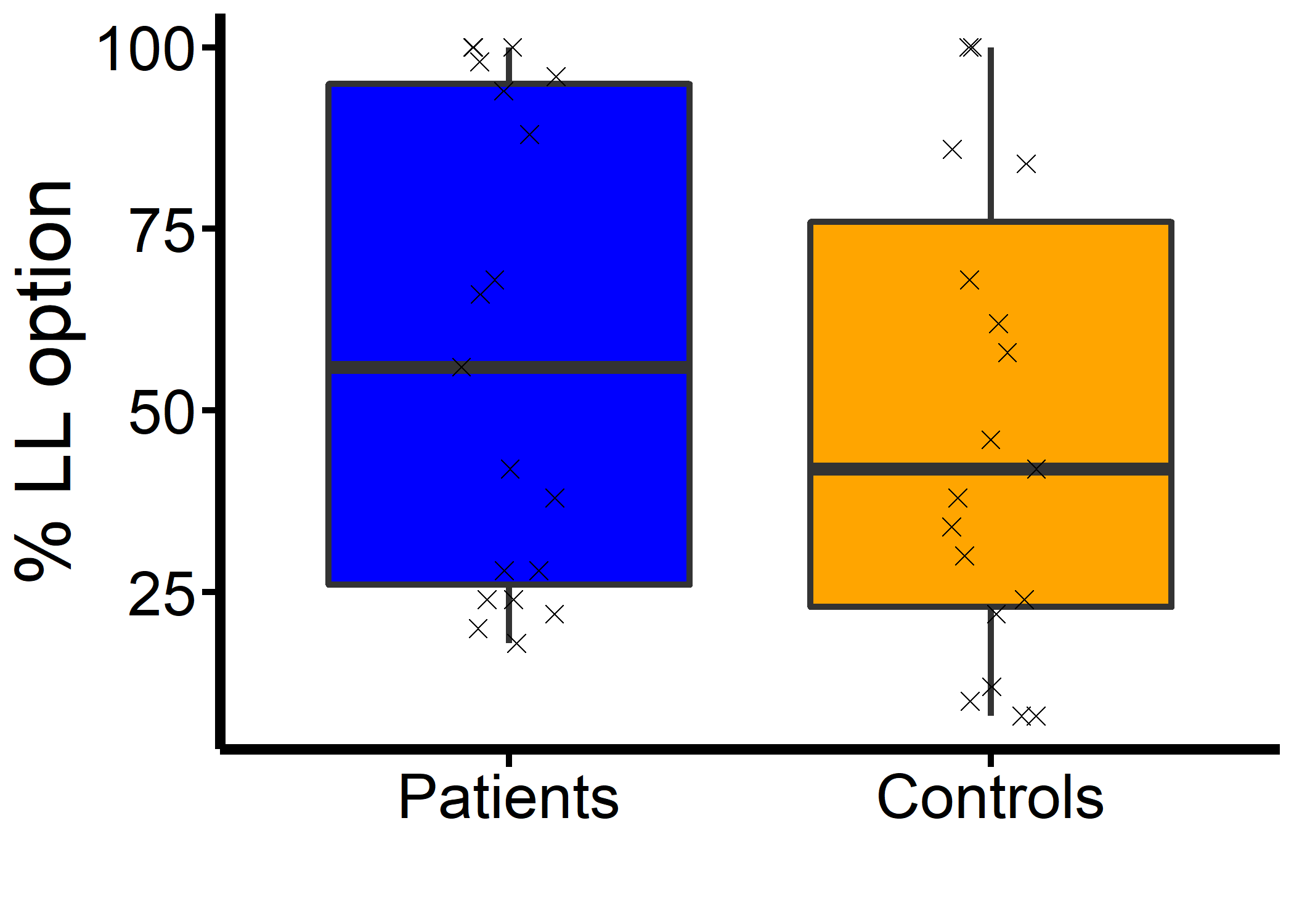

Supplement: S2 Fig — LL, larger but later. (TIF) [file pone.0253620.s002.tif]

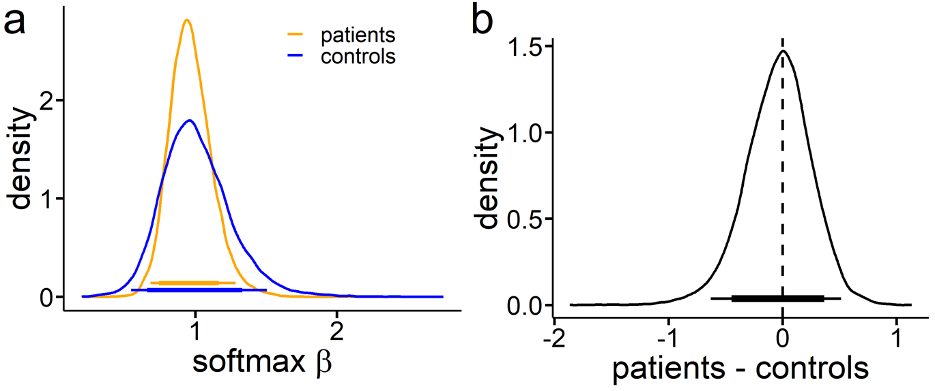

Supplement: S3 Fig — A: Softmax β in adolescent patients with TS vs. controls. Group level hyperparameter distributions of the inverse temperature parameter softmax β revealed no group differences between patients (orange) and controls (blue). B: Difference distribution of controls—patients with TS. Thin and thick colored (a) and black (b) bars indicate the 95% and 85%. TS, Tourette syndrome. (TIF) [file pone.0253620.s003.tif]

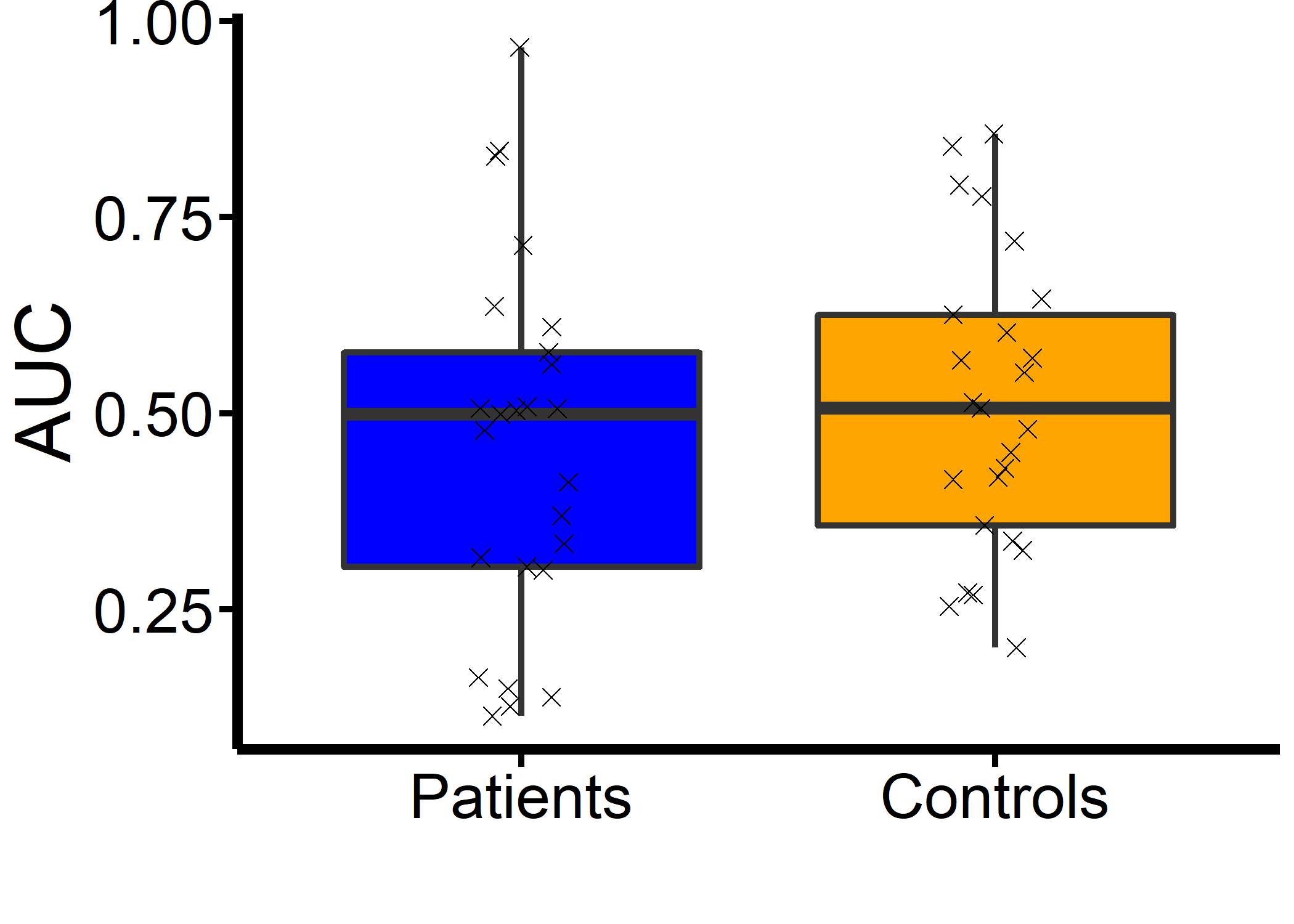

Supplement: S4 Fig — TS, Tourette syndrome. (TIF) [file pone.0253620.s004.tif]

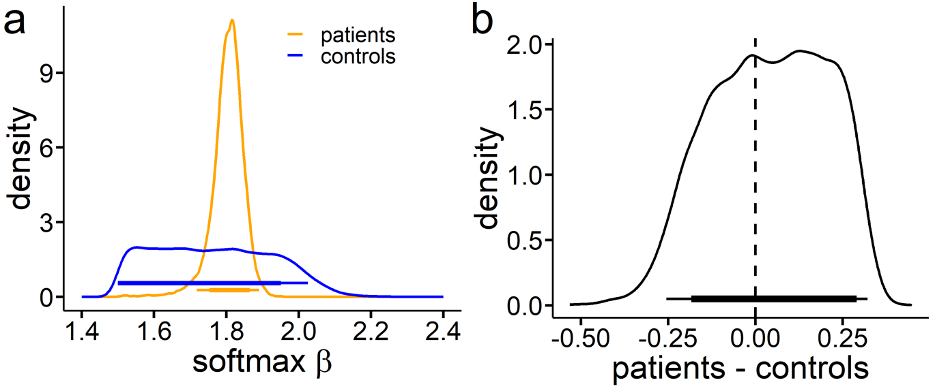

Supplement: S5 Fig — A: Softmax β in adult patients with TS vs. controls. Group level hyperparameter distributions of the inverse temperature parameter softmax β revealed no group differences in the mean of the posterior or a shift in either direction between patients (orange) and controls (blue). However, variance was increased in controls indicating higher interindividual differences in decision noise. B: Difference distribution of controls—patients with TS. Thin and thick colored (a) and black (b) bars indicate the 95% and 85% highest density intervals respectively. TS, Tourette syndrome. (TIF) [file pone.0253620.s005.tif]
