# Supplementary material for: Temporal discounting in adolescents and adults with Tourette syndrome
Source: PLoS One. 2021 Jun 18;16(6):e0253620. doi: 10.1371/journal.pone.0253620 (PMC8213148; doi:10.1371/journal.pone.0253620)
Supplement: S1 Table — Discount-rate (k) parameters were estimated in logarithmic space due to parameter stability. Softmax β values were estimated in standard-normal space for the same reason. (DOCX) [file pone.0253620.s006.docx]

| **Model** | **Parameter** | **Prior** |
| --- | --- | --- |
| Hyperbolic | *k* | uniform (-20,3) |
|  | softmax *β* | uniform(-4,4) |
| Exponential | *k* | uniform(-2,4) |
|  | softmax *β* | uniform(-4,4) |
