# Supplementary material for: Temporal discounting in adolescents and adults with Tourette syndrome
Source: PLoS One. 2021 Jun 18;16(6):e0253620. doi: 10.1371/journal.pone.0253620 (PMC8213148; doi:10.1371/journal.pone.0253620)
Supplement: S2 Table — We report our exploratory analysis on discount-rate and questionnaire data. Scores are spearman correlation coefficients (p-value) not corrected for multiple comparisons. TS, Tourette syndrome. (DOCX) [file pone.0253620.s007.docx]

|  | **Adolescent patients with TS (*n*=19)** | **Controls (*n*=19)** |
| --- | --- | --- |
| Subscale | median(*k*) | median(*k*) |
| SBB Attentional | 0.30 (0.22) | -0.001 (1.00) |
| SBB_Motor | 0.40 (0.10) | 0.21 (0.41) |
| SBB Impulsive | -0.08 (0.76) | 0.23 (0.35) |
| SBB ADHD | 0.13 (0.6) | 0.13 (0.60) |
| Age | -0.16(0.50) | -0.09(0.71) |
| CY-BOCS | -0.39(0.10) | - |
| YBGTSS | -0.03(0.91) | - |
