# Supplementary material for: Temporal discounting in adolescents and adults with Tourette syndrome
Source: PLoS One. 2021 Jun 18;16(6):e0253620. doi: 10.1371/journal.pone.0253620 (PMC8213148; doi:10.1371/journal.pone.0253620)
Supplement: S3 Table — We report our exploratory analysis on discount-rate and questionnaire data. Scores are spearman correlation coefficients (p-value) not corrected for multiple comparisons. TS, Tourette syndrome; BDI, Becks depression inventory; OCI-R, Obsessive-Compulsive Inventory-Revised; TS, Tourette syndrome; WURS-k, Wender-Utah-Rating-Scale. (DOCX) [file pone.0253620.s008.docx]

|  | **Adult patients with TS (*n*=25)** | **Controls (*n*=25)** |
| --- | --- | --- |
| **Questionnaire** | **median(*k*)** | **median(*k*)** |
| **WURSK-k** | 0.13 (0.54) | 0.29 (0.15) |
| **OCI-R** | 0.17 (0.41) | 0.32(0.11) |
| **BDI** | 0.07 (0.73) | 0.18(0.39) |
| **Age** | -0.39 (0.05) | -0.40(0.04) |
